# Genome-wide analysis and characterization of GRAS family in switchgrass

**DOI:** 10.1080/21655979.2021.1972606

**Published:** 2021-09-03

**Authors:** Xiaoqin Wang, Guixia Li, Yajing Sun, Zhongyu qin, Pengcheng Feng

**Affiliations:** a Department of Anesthesiology, Changzhi Medical College, Changzhi, Shanxi, China; b Department of Basic Medicine, Changzhi Medical College, Changzhi, Shanxi, China; c Department of Biochemistry and Molecular Biology, College of Life Sciences, Jilin University, Changchun, Jilin, China

**Keywords:** GRAS, *Panicum virgatum*, genome-wide analysis, gene family, gibberellin

## Abstract

*Panicum virgatum*, a model plant of cellulosic ethanol conversion, not only has high large biomass and strong adaptability to soil, but also grows well in marginal soil and has the advantage of improving saline-alkali soil. GRAS transcription factor gene family play important roles in individual environment adaption, and these vital functions has been proved in several plants, however, the research of GRAS in the development of switchgrass (*Panicum virgatum*) were limited. A comprehensive study was investigated to explore the relationship between GRAS gene family and resistance. According to the phylogenetic analysis, a total of 144 GRAS genes were identified and renamed which were classified into eight subfamilies. Chromosome distribution, tandem and segmental repeats analysis indicated that gene duplication events contributed a lot to the expansion of GRAS genes in the switchgrass genome. Sixty-six GRAS genes in switchgrass were identified as having orthologous genes with rice through gene duplication analysis. Most of these GRAS genes contained zero or one intron, and closely related genes in evolution shared similar motif composition. Interaction networks were analyzed including DELLA and ten interaction proteins that were primarily involved in gibberellin acid mediated signaling. Notably, online analysis indicated that the promoter regions of the identified PvGRAS genes contained many cis-elements including light responsive elements, suggesting that PvGRAS might involve in light signal cross-talking. This work provides key insights into resistance and bioavailability in switchgrass and would be helpful to further study the function of GRAS and GRAS-mediated signal transduction pathways.

## 1. Introduction

*Panicum virgatum*, an annual plant helianthus, is a promising feedstock for value-added applications due to its high productivity, potentially low requirements for agricultural inputs and positive environmental impacts [1,2]. Specifically, as a model plant of cellulosic ethanol conversion, it not only has high photosynthetic capacity, developed root system, rich cellulose content, rapid growth, large biomass and strong adaptability to soil, but also grows well in marginal soil and has the advantage of improving saline-alkali soil [3,4].

It has to be mentioned that transcription factors (TFs) are key signaling components against abiotic stresses for plants [5]. Therefore, in the molecular genetic field, identification of plant key TFs to explore signal transduction and abiotic stresses can yet be regarded as an important topic. Translational genomics can be considered as a good research approach to gene identification in switchgrass [6,7].

The GRAS, an important transcription factor gene family in plant, was denominated after Gibberellic acid insensitive (GAI), Repressor of GA1 (RGA) and Scarecrow (SCR) which were the first three functionally identified members [8]. GRAS genes have been considered as plant-specific transcription regulator family and play critical roles in development and signaling [8–10]. BrLAS-overexpressing Arabidopsis plants displayed significantly enhanced drought resistance [11]. Overexpress of ZmGRAS20 can led to decreased starch content [12,13]. Previous research shows overexpressing SiGRAS40 in transgenic tomato plants can be more tolerant of drought and salt stress [14]. By far, GRAS gene family has been explored in several plant species, including *Populus, Arabidopsis*, rice, pepper, tobacco, *Prunus mume*, tomato, *Juglans regia*, and so on [15–20].

The research that performed on switchgrass in genome wide could infer as many GRAS genes as possible could provide enough genetic resources for our basic research such as cultivate drought resistance varieties. and the potential functions of specific genes analyzed by phylogenetic tree analysis and homology comparison could screen genes purposefully. In the present paper, GRAS genes in switchgrass have been identified comprehensively. The phylogenetic relationship, gene structure, duplication event, cis-element and expression profiling of the switchgrass GRAS genes were systematically analyzed. Finally, interaction network analysis was used to study how proteins with similar functions are associated with which proteins. qRCR analysis demonstrated that PVGRAS17 and PVGRAS103 might be used for drought and salt tolerance breeding respectively. The results of our study would not only provide key insights into the evolution, classification, and function of GRAS proteins, but provide new insights and new ideas for genetic research in *Panicum virgatum*. Furthermore, it would be helpful to with drought resistance and salt stress in switchgrass.

## 2. Materials and methods

### 2.1. Identification and annotation of GRAS genes in switchgrass

The latest versions of the genome annotations and genome sequences of *Panicum virgatum* were downloaded from the phytozme database. The GRAS transcription factors of *Arabidopsis thaliana* were downloaded from TAIR. The genome annotations and genome sequences of *Oryza sativa* were downloaded from EnsemblPlants. All GRAS proteins from *Panicum virgatum* were acquired by TBtools [21]. All the potential GRAS genes were further confirmed by BLASTP. Molecular weight (MW) and isoelectric point (pI) were predicted by ProtParam online toolkits ExPASy [22].

### 2.2. Phylogenetic analysis and classification of the PvGRAS gene family

Multiple alignments were performed using Muscle in default parameters based on the GRAS protein sequences from *Arabidopsis thaliana* and switchgrass. MEGA6.06 was adopted to build a phylogenetic tree by Neighbor-joining method (NJ) with the 1,000-time bootstrap tests [23,24].

### 2.3. Detection of gene structures and conserved motifs

TBtools (Version 0.6696) was used to show exon-intron structure of PvGRAS by submitting the genome annotation profile and gene IDs [25]. The program MEME/MASAT was used for GRAS conserved motif analysis [26], which the parameters were set as the default value except for the option ‘MEME should find’ which was set to 15. The conserved motif structures were shown by TBtools.

### 2.4. Chromosomal locations, interaction network and duplication events analysis

The chromosomal positions of all GRAS members were confirmed using the *Panicum virgatum* genome annotation file. The tandem and segmental gene duplications were identified by Circos and TBtools as previously described [27,28]. Protein interaction network prediction was performed by STRING [29] and visualized by Cytoscape v3.7 [30]. The syntenic analysis map was generated to show the synteny relationship of the orthologous genes extracted from *Panicum virgatum* and *Oryza sativa*, following the non-synonymous (Ka) and synonymous (Ks) of each duplicated gene pair were calculated by TBtool [31]. psRNATarget (http://plantgrn.noble.org/psRNATarget/ analysis) was performed to predict miRNA of all PvGRAS genes.

### 2.5. PvGRAS promoter analysis

cis-elements play essential roles in the regulation of plant growth and adaption to the environment. To investigate cis-acting elements in the promoter regions of PvGRAS, the sequence 2000bp upstream of the start codon was extracted and used as a hypothetical promoter. and then submitted them to online PlantCARE tool (http:// bioinformatics.psb.ugent.Be/webtools/plantcare/html/) to predict cis-acting elements and identify the putative cis-element [32]. The elements with same functions were arranged using EXCEL. And TBtools software was applied to analyze the final results.

### 2.6. GO enrichment analysis

Pannzer2 (http://ekhidna2.biocenter.helsinki.fi/sanspanz/), an online tool, was performed to conduct Gene Ontology (GO) enrichment analysis, including molecular function (MF), biological process (BP) and cellular component (CC), to explore the rule of 144 PvGRAS. All Amino acid sequences were submitted in fasta format. Bioinformatics (http://www.bioinformatics.com.cn/) was used to annotate the GO terms.

### 2.7. Expression profile of PvGRAS genes

The Uni-transcript IDs of the PvGRAS were identified in the PviUTs database [33]. The integrated expression data was obtained by searching the Switchgrass Gene Expression Atlas (PviGEAs) [33,34]. The results were graphically presented in a heatmap format with log fold change after value normalization. For the heat-responsive transcription analysis of the PvGRAS, data from the ArrayExpress repository under the accession number E-MTAB-1897 [35] were retrieved. A total of 144 PvGRAS retrieved from the array data were presented in a heatmap with log_2_ fold change after value normalization.

### 2.8. Plant growth and stress treatment

Switchgrass seedlings were obtained from Shandong Luhong Flower Gardening Company. Plants are grown at room temperature of 25 ± 1°C, 16 h light/20 ± 1°C, 8 h night and watered regularly and quantitatively. Six weeks old plants with similar growth conditions were selected to mimic salt, drought abiotic stress, which included 25% polyethylene glycol (PEG) 6000 for 0, 3, 6, 12 and 24 h; 300 mM NaCl for 0, 3, 6, 12 and 24 h. No less than 3 plants were selected per abiotic stress treatment.

### 2.9. Expression analysis of the PvGRAS genes by qPCR

Total RNA of switchgrass was extracted using the RNA extraction kit (RNAiso Plus, 9180Q, Takara) and treated with RNase free DNase I. The cDNA was pretreated with Reverse Transcriptase M-MLV (RNase H-) (2641A Takara) and Random Primer (nonadeoxyribonucleotide mixture; pd (N) 9) (3802 Takara). The CDS sequences of PvGRAS genes were obtained based genome sequence and genome annotation information. The primer pairs were designed using Primer 5. eEF-1α gene were used as internal control. And the detailed information of the primers used in qPCR was listed in **Supplementary Table S1**. qPCR was performed using TB Green Premix Ex Taq (Tli RNaseH Plus) (RR420A Takara) and repeated three times. By adjusting temperature or reseting the primers, until a dissolution curve with a single peak is observed. LineGene K Plus Fluorescence Quantitative PCR Detection System (FQD-48A) was used to detect the expression level of GRAS genes, The data were analyzed by the 2−(^ΔΔCt^) method and SPSS 26. Finally, EXCEL was used to display the gene expression map.

## 3. Results

### 3.1. Identification and annotation of GRAS genes in switchgrass

**Figure 1.**
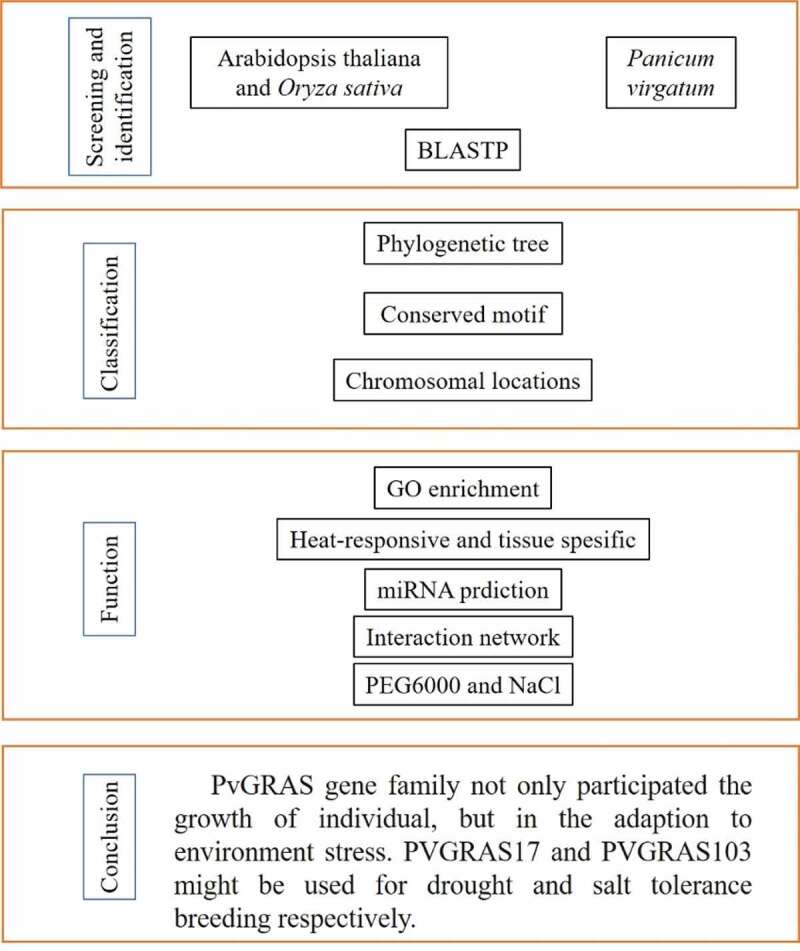
A flow chart based on the content

A flow chart was drawn to facilitate readers to understand the writing ideas in Figure 1. 189 GRAS candidate genes were identified by local BLASTP, following 144 GRAS genes were identified by SMART of which 9 genes were re-annotated. GRAS genes were renamed from PvGRAS1 to PvGRAS144 according to gene location on chromosome. The protein molecular weight (MW) of identified members ranged from 25.6704 KDa to 206.2645 KDa, and the isoelectric point (PI) ranged from 4.70 to 9.91. The largest GRAS protein was PvGRAS143 which MW was more than 200 KDa. Detailed information could be available in **Supplementary Table S2**.

### 3.2. Phylogenetic analysis and classification of the PvGRAS gene family

**Figure 2.**
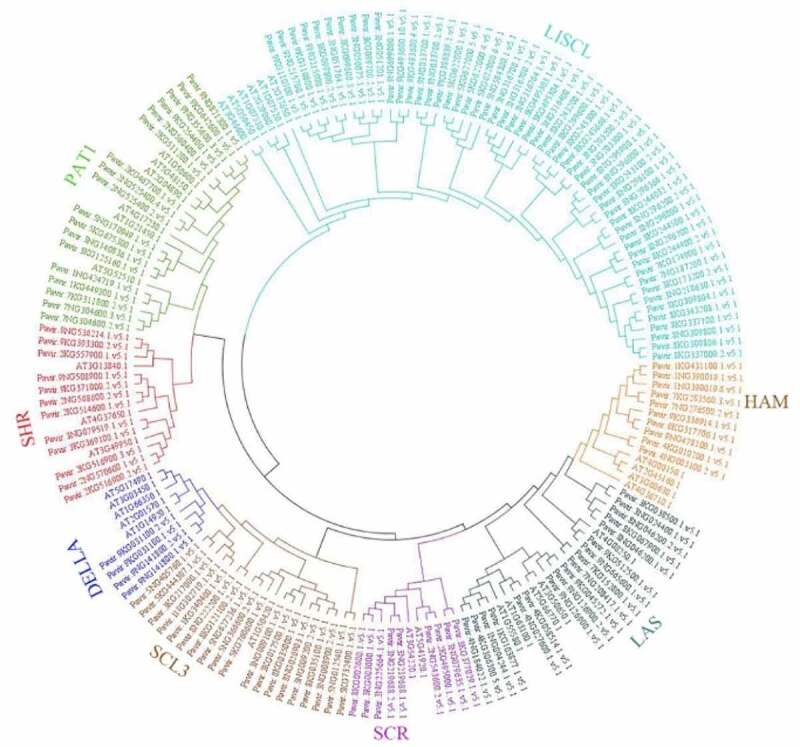
Phylogenetic relationships of GRAS genes. The phylogenetic tree (neighbor joining) was constructed using all of the candidate GRAS proteins in *P. virgatum* (Pv: 144) and *A. thaliana* (At: 31) which were classified into 8 subfamilies. The branches sharing the same color are in the same subfamily

A phylogenetic tree was constructed contains 144 PvGRAS amino acid sequences as well as 32 *Arabidopsis* GRAS genes to reconstruct the evolutionary relationship (Figure 2). The PvGRAS genes were classified into 8 subfamilies, which were designated following earlier studies [36], namely, HAM, DELLA, SCL3, SCR, LAS, SHR, PAT1 and LISCL according to the previous classification of GRAS families. The GRAS unevenly distributed in different subfamilies. For example, the LISCL subfamily containing 60 GRAS members as the largest subfamily, including 54 switchgrass GRAS genes and 6 *Arabidopsis* GRAS genes, whereas DELLA subfamilies were the smallest subfamily contained only 8 GRAS members (4 PvGRAS, 4 AtGRAS).

### 3.3. Analysis of gene structures and conserved motifs

**Figure 3.**
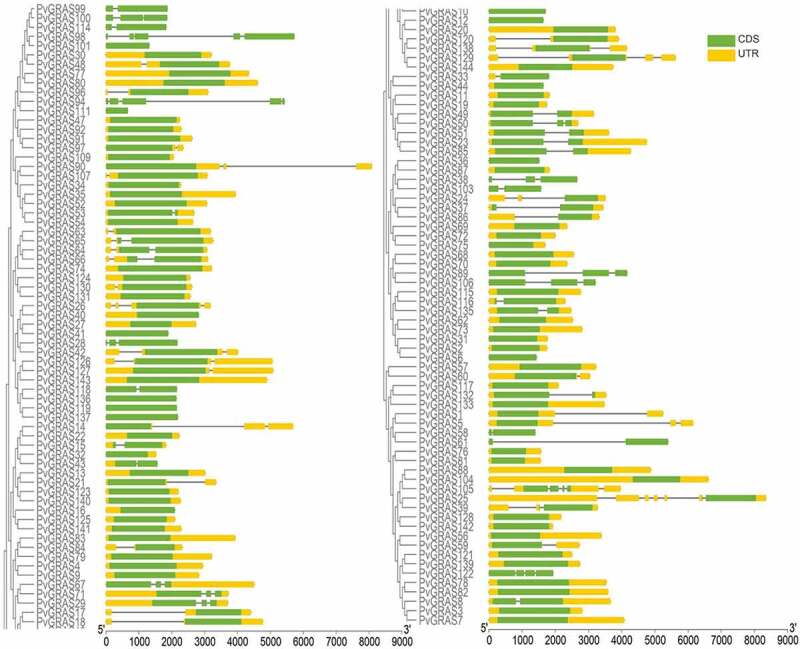
Exon-intron structure of switchgrass GRAS genes. Orange boxes indicate non-coding regions (UTR), green boxes indicate coding sequences (CDS), and black lines indicate introns

**Figure 4.**
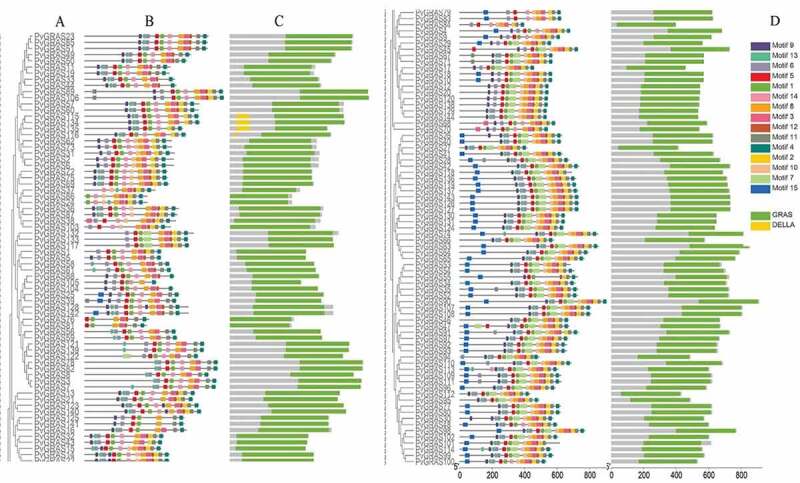
Structure analysis of PvGRAS genes. (a) Phylogenetic tree of GRAS proteins in switchgrass. (b) Distribution of motifs of PvGRAS proteins. (c) The conserved domains in PvGRAS genes. The length of the nucleic acid sequences is indicated by the length of the line, and the positions of the color blocks on the lines are the position of the motifs on the nucleic acid sequence. (d)Fifteen colors represent different motifs and the green color represent GRAS domains and yellow corresponding DELLA domains

All of the PvGRAS genes were used to analyze the distribution of exons and introns to explore the characteristics of the GRAS genes (Figure 3). The analysis of conserved motifs among sequences was predicted to understand the evolutionary relationships. The C-terminus of GRAS proteins were proved to be highly conserved in terms of sequence homology (Figure 4). The GRAS domain, a typical structure, was identified in all PvGRAS. Fifteen different motifs were identified and used to understand each group. Members of the same group were highly similar as far as motif composition but differed from other groups. The common motif in all PvGRASs was motif 4 and motif 8. It should be noticed that PvGRAS55 lacked several motifs compared to adjacent branches although it was re-annotated when *Orayza sativa* was regarded as gene-finding parameter.

### 3.4. Chromosomal locations, duplication events, synteny analysis of PvGRAS genes analysis

**Figure 5.**
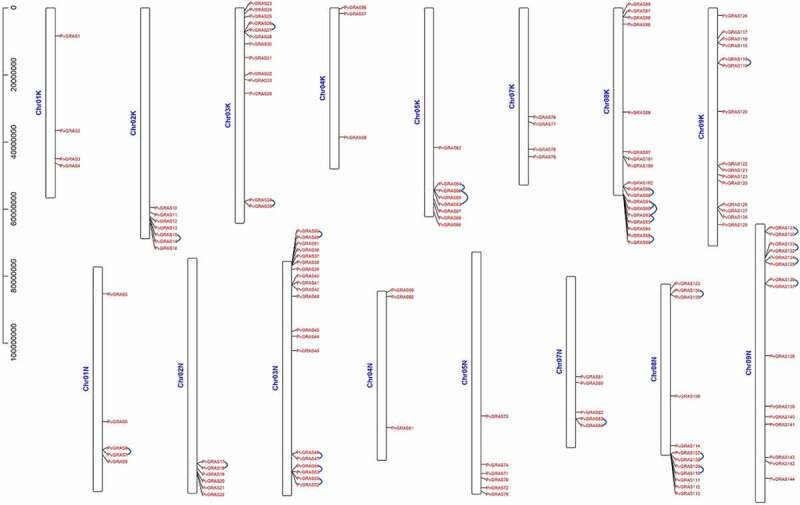
Positions of GRAS gene family members on the switchgrass chromosomes. The leftmost is the chromosome length ruler. The chromosomes were represented by the bars in the figure, and the length represented the size of the chromosomes. The GRAS genes were marked in red at the corresponding position on the chromosome. Genes in tandem repeats are shown beside the blue lines

In total, Most of the GRAS genes (66%) were found on Chromosome 03 K&N, 08 K&N, 09 K&N (Chr03K&N, Chr08K&N, Chr09K&N), by contrast, Chr04 K&N contained only six genes (4%) (Figure 5). Nine PvGRAS genes were distributed on Chr01K&N and Chr07K&N. The GRAS genes on Chr02K&N, Chr03K&N, Chr05K&N, Chr08K&N were almost gathered next to each other near the two sides, nevertheless, GRAS genes on Chr09K&N were uniformly distributed on the whole chromosome. Distribution on chromosomes of PvGRAS genes was unevenly and irregular.

**Figure 6.**
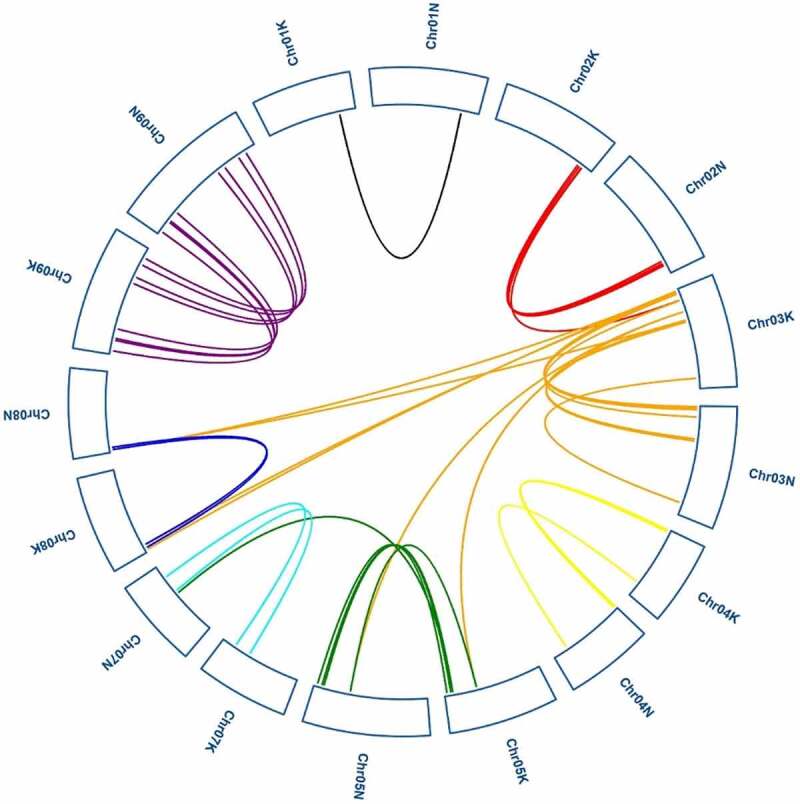
Synteny analysis of PvGRAS genes. Black, red, orange, yellow, green, cyan, blue and purple lines were shot from 1–9 chromosomes (no include 6), respectively. lines indicate all synteny gene pairs in the switchgrass genome

We analyzed tandem and segmental duplications of the PvGRAS transcription factors. Among the 144 PvGRAS genes, twenty-five pairs were found in tandem repeats where the largest cluster was on Chr08 K&N. The results of duplicated gene pairs showed all Ka/Ks ratios were less than 0.8, suggesting these genes went through purifying selection pressure not positive selection in the course of evolution. Gene duplication events were investigated to grope the model of evolution of the GRAS gene family of switchgrass genome. Analysis of switchgrass GRAS family genes revealed forty paralogous gene pairs existed in switchgrass GRAS family genes (Figure 6). The ratio of nonsynonymous substitution per site (Ka) to synonymous substitution rate per site (Ks) was used to show the molecular evolution of PvGRAS, the ratios of Ka/Ks of all the tandem amplification and segmental duplication were showed on **Supplementary Table S3**.

**Figure 7.**
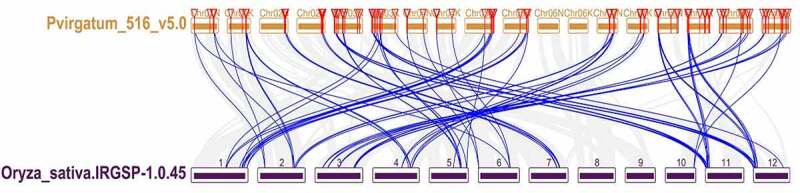
Synteny analysis of the GRAS genes between switch and Oryza sative. Gray lines in the background indicate the collinear blocks within switchgrass and Oryza sative, while blue lines highlight the syntenic PvGRAS gene pairs

Analysis of collinearity relationship can provide orthologous relationships between different species. For purpose of the evolutionary relationship of GRAS gene between *Oryza sativa* and *Panicum virgatum*, syntenic analysis was carried out for two plants (Figure 7). Through synteny analysis, many collinear blocks were found between rice and switchgrass. And sixty-six switchgrass GRAS genes showed pairwise synteny with genes in the rice genome.

### 3.5. miRNA prediction

**Figure 8.**
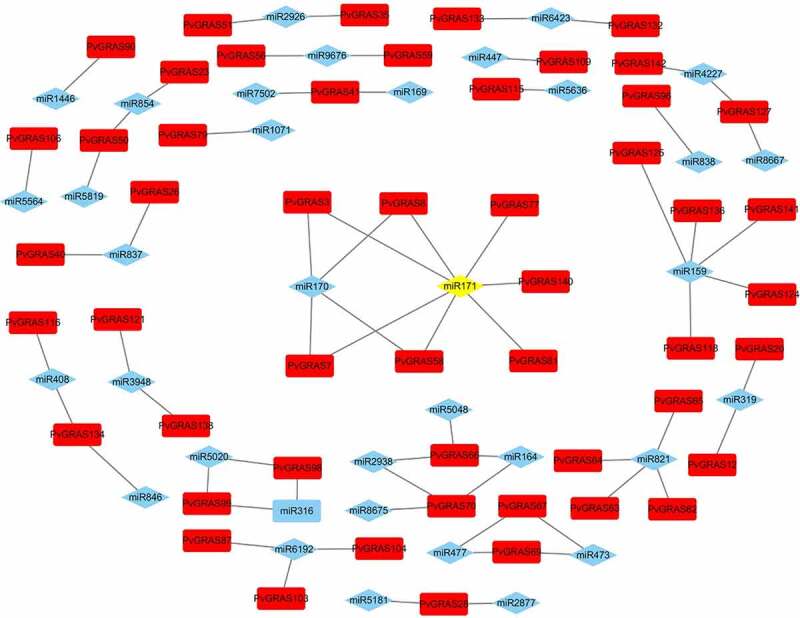
The interaction between miRNA and PvGRAS. Red blocks: PVGRAS, blue: miRNA and yellow: miR171. Lines between the squares indicated that they could interact with each other

In the field of human diseases, the roles of miRNA have been well-researched, however, the knowledge of miRNA in plants is fewer. Previous research had reported GRAS genes could be regulated by miRNA171. Our results showed that 7 PvGRAS genes from HAM subfamily (PvGRAS140, PvGRAS3, PvGRAS58, PvGRAS7, PvGRAS77, PvGRAS8, PvGRAS81) were complementary to miRNA171. In this study, 38 miRNAs were found to bind to 56 PvGRAS genes (**Supplementary Table S4**). The results were shown in (Figure 8) by Cytoscape.

### 3.6. PvGRAS promoter analysis

**Figure 9.**
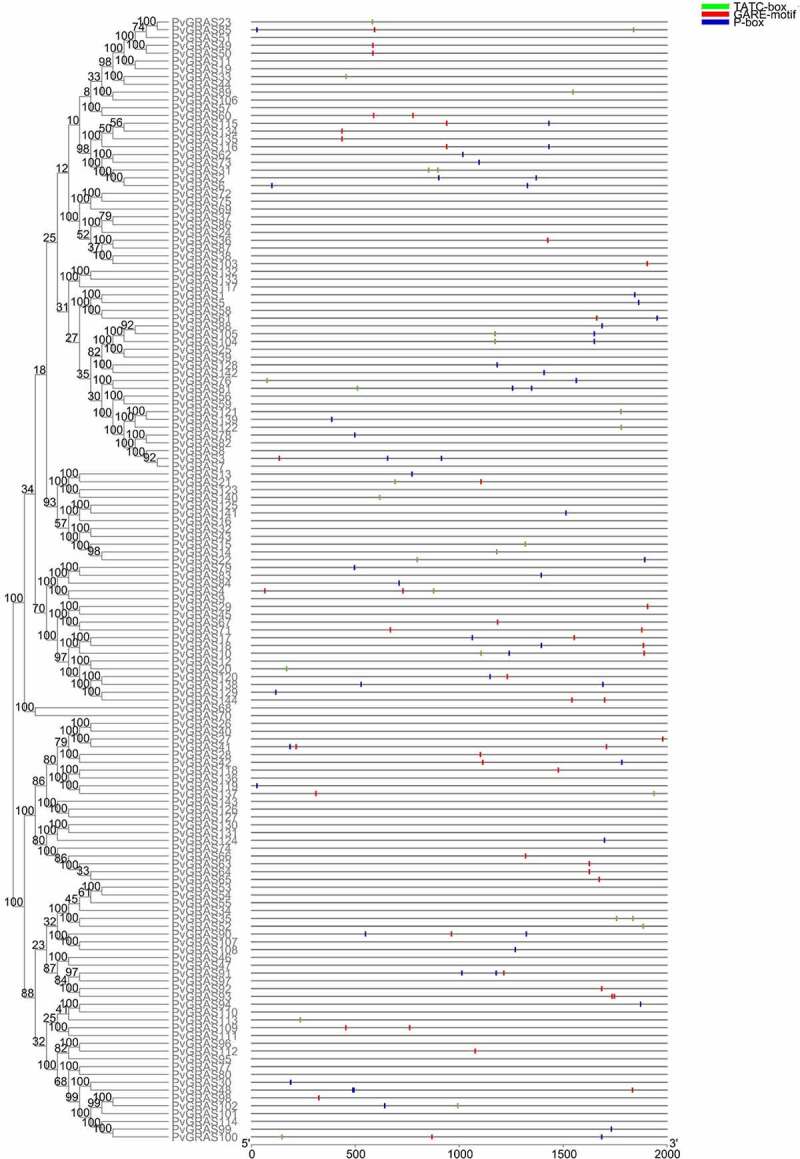
Cis-elements about GARE-motif, TATC-box and P-box in PvGRAS promoters analyzed by plantCARE

Forty-seven cis-elements were identified in the −2kb region upstream of the transcription initiation site of the GRAS gene, of which twenty-eight were related to light responsive. Five elements of kinds were related to hormone responsiveness, seven elements were related to stress responses, remaining elements respected for different roles. It was worth noting that the number of light response elements was the most, almost distributed in every PvGRAS, with at least two elements. And more than half of PvGRAS genes contained hormone (IAA, MeJ, SA and ABA) elements with no more than three. More specifically, hormone response elements were involved in GA (Figure 9 and **Supplementary Table S5**). Four cis-elements (dehydration, low-temperature, salt, drought anaerobic induction) related to abiotic stress response and several tissue developments (meristem expression, root or seed-specific, cell cycle regulation and wound cis-elements) related cis-elements were found in promoter regions. This suggested that the PvGRAS genes might play important parts in the growth and development of switchgrass and respond to external stress.

### 3.7. Interaction network of PvGRAS proteins

**Figure 10.**
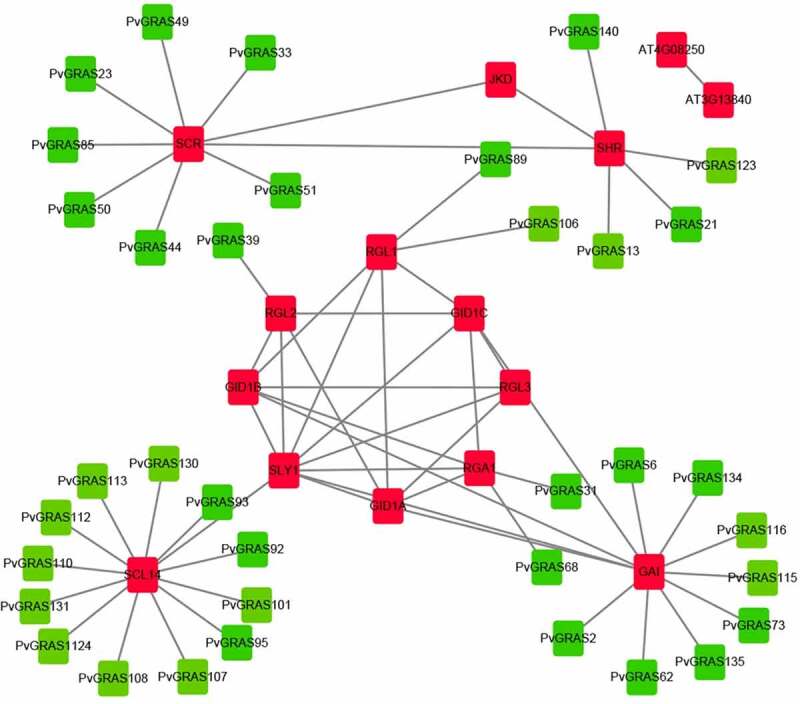
Functional interaction networks of PvGRAS proteins in switchgrass according to orthologs in *Arabidopsis*. Red: GRAS proteins from *Arabidopsis*, green: GRAS proteins from switchgrass. The lines between red block represented that they could interaction with each other, the lines between red and green block represented the homologous relationship

A protein interaction network was constructed for PvGRAS based on the homology analysis between PvGRAS protein and *Arabidopsis* using the STRING database with the highest bit score. Thirty-six GRAS proteins and fifteen proteins in *A. thaliana* were used to build interactive network map (Figure 10). SCL14 (scarecrow-like protein 14) affects the transcription of stress-responsive genes by binding to SLY1 that is an essential component of the SCF-type E3 ligase complex, SCF (GID2), a complex that positively regulates the gibberellin signaling pathway.

### 3.8. GO enrichment analysis

**Figure 11.**
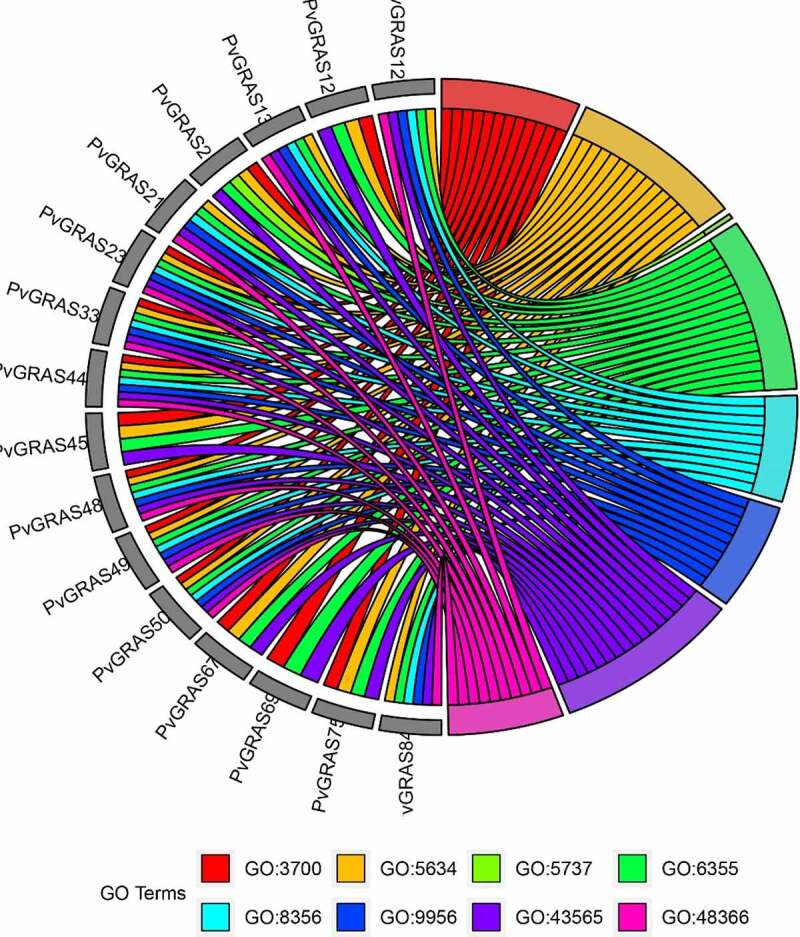
Top 7 GO enrichment analysis results were shown. GO3700: DNA-binding transcription factor activity, GO5634: nucleus, GO5737: cytoplasm, GO6355: regulation of transcription, DNA-templated, GO8356: asymmetric cell division, GO9956: radial pattern formation, GO43565: sequence-specific DNA binding, GO48366: leaf development

Seventy-one terms, containing 44 BP, 4 CC and 23 MF, were enriched. The main enriched terms include DNA-binding transcription factor activity (GO:3700), nucleus (GO:5634), regulation of transcription, DNA-templated (GO:6355) and sequence-specific DNA binding (GO:43565). Several terms, such as regulation of secondary shoot formation (GO: 2,000,032), regulation of seed dormancy process (GO: 2,000,033), root development (GO: 48,364), leaf development (GO: 48,366) and negative regulation of seed germination (GO: 10,187), that regulate the development of individual organs and tissues have also been enriched. Seven hormone-related terms, response to ethylene (GO: 9723), response to abscisic acid (GO: 9737), response to gibberellin (GO: 9739), gibberellic acid mediated signaling pathway (GO:9740), hormone-mediated signaling pathway (GO: 9755), salicylic acid mediated signaling pathway (GO: 9863) and jasmonic acid mediated signaling pathway (GO: 9867) were detected. The detailed information of GO enrichment was shown in **Supplementary Table S6**. The corresponding relationships between top 16 gene names and the top 8 of GO terms are shown in Figure 11.

### 3.9. Expression profiles of PvGRAS genes

**Figure 12.**
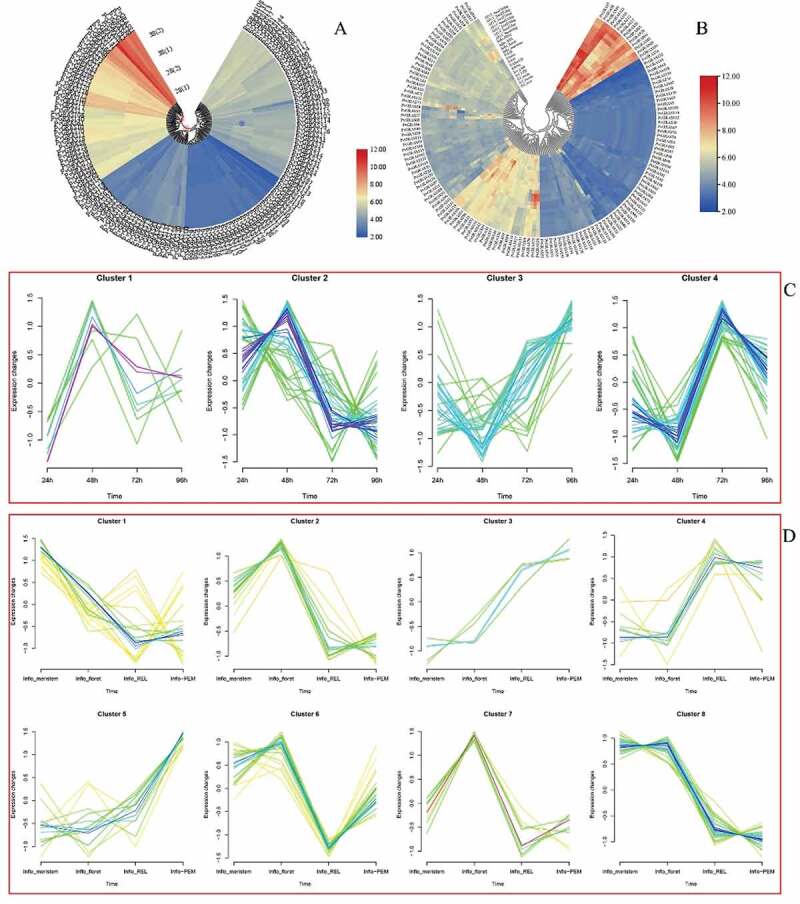
GRAS genes expression pattern analysis. a. genes expression in different temperature. b. The PVGRAS genes special expression profiles in different tissues. c. Time-dependent cluster analysis. a Clustering according to changes in expression levels at different time (24, 48, 72, 96 h) points during seed germination. b Clustering according to changes in different development stages (meristem initiation, floret development, Rachis and brach elongation, Panicle emergence) during inflorescence development

Here, both switchgrass Affymetrix array and PviUT database were used to pool out expression profiles of PvGRAS genes. The normalized data from E-MTAB-1897 was used to analyze the express level of PvGRAS genes under the heat. Through the clustering of expression levels, GRAS genes can be divided into four categories under the stimulation of temperature. forty PvGRAS genes were higher expression, whereas one hundred-four PvGRAS genes were lower expression and the expression levels of most PvGRAS genes are not significantly different between 28°C Celsius and 38°C. The expression level of fourteen genes (PvGRAS65, 29, 82, 83, 89, 20, 12, 17, 106, 111, 16, 91, 109 and 27) were increased, however eight genes (PvGRAS70, 11, 18, 104, 42, 44, 54 and 114) were decreased. The detailed information was shown in Figure 12(A).

PviUT was used to analyze the gene expression level in the development process. The expression patterns of all PvGRAS genes were pooled out in 21 differential tissues or developmental stages. According to gene expression level, GRAS genes can be divided into four modules. Fourteen genes were high expression in all tissue and developmental stages. Twenty-three genes were moderate expression, the other two modules were low expression (Figure 12(B)). In the seed and flower tissues, the expression level of almost all PvGRAS genes shown significant differences. Spatial expression analysis of gene expression, the gene showed differential expression in different tissues. In the part of Inflorescence development analysis, similar conclusions were also obtained. Furthermore, The R package ‘Mfuzz’ was used to explored the time-dependent cluster analysis. According to the expression pattern of GRAS genes in seed germination, four modules were clearly identified. Eight parts were separated in inflorescence development (Figure 12(C) and (D)).

### 3.10. The rule of six PvGRAS genes under the abiotic stress

**Figure 13.**
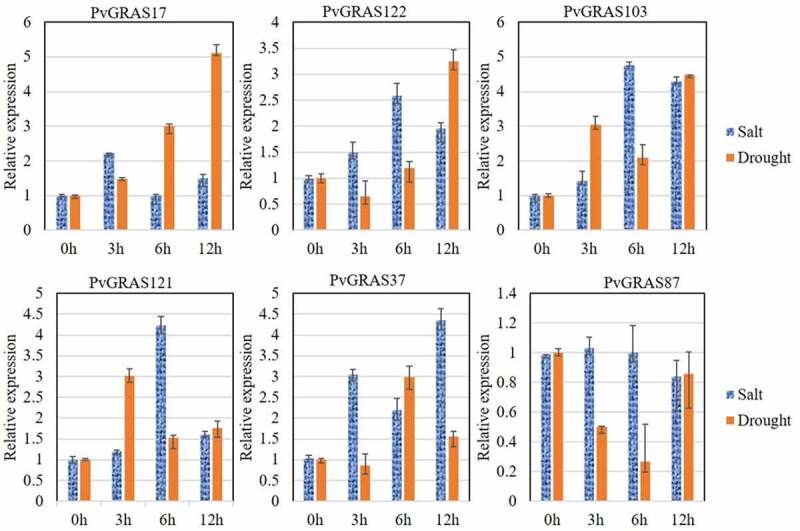
The PVGRAS genes special expression under drought and salt

Drought and salt are two important abiotic stresses that affect plant growth. In order to investigate the response of GRAS gene family in the drought and salt, q-PCR was performed to detect the change of six random selected GRAS genes after the treatment of PEG6000 or NaCl at 0 h, 3 h, 6 h and 12 h. The expression level of these six genes were changed significantly after the treatment of PEG6000 or NaCl. the relative expression of PvGRAS103 were increased by 4.8 times at 6 h under salinity stress, however, the expression of PvGRAS87 was not obvious changed. That result shown that different genes have various response strengths to the same stress. Similar results were observed under the stress of drought. In addition, the same gene exhibits different expression pattern under different stresses. The detailed information was shown in Figure 13.

## 4. Discussion

Many studies have shown that transcription factors, including the GRAS gene family, play a vital role in resisting biotic, abiotic, and individual growth and development. Studies about the GRAS gene family of some model plants and crops have shown that the GRAS gene family has undergone special amplification. But the knowledge of GRAS genes in switchgrass, a potential biofuel crop, is limited. Here, the numbers of GRAS gene family in switchgrass, gene structure, conserved motifs, and chromosomal locations in switchgrass were identified and analyzed.

### 4.1. Switchgrass has a large number of unique GRAS genes under diversifying selection

To identify GRAS genes in switchgrass, all the CDS sequences were obtained by TBtools using genome annotation and genome sequence, then these sequences were translated to protein sequences. The local BLASTP search was performed using the amino acid sequences of *Arabidopsis* GRAS members as queries. According to the characteristics of GRAS family, the candidates were excepted which the length was out of the range from 350 to 820 aa. Then, resulted hits were subsequently subjected to SMART analysis. A total of 144 GRAS genes in the switchgrass genome were identified and could be divided into eight subgroups. The number of PvGRAS was the largest compared to previous reports, including those in rice (57), *Arabidopsis thaliana* (32) [36], pepper (*Capsicum annuum*) (50) [16], *Juglans regia* (52) [19], *maize* (86) [37], and tomato (*Solanum lycopersicum*) (53) [15,38,39], potato (*Solanum tuberosum*) (52) [24]. This large number of GRASs in switchgrass could be due to the recent polyploidization events (less than 2 million years ago) between two closely related diploid progenitors of switchgrass [40,41]. Moreover, according to the established phylogenetic tree with all GRAS genes of switchgrass and 32 *Arabidopsis* GRAS genes, a high percentage of PvGRAS were outside of the subgroups assigned for *Arabidopsis* GRAS, reflecting the divergence and expansion of specific groups of GRAS genes in switchgrass.

The tetraploid switchgrass is disomic inheritance with two sub-genomes likely originated from a polyploidization event between closely-related diploids. This disomic inheritance displays more opportunities than polysomic inheritance which promotes the duplicated genes to undergo divergence and development of new functions [41,42]. An important finding was that most PvGRAS genes (78%) contained just one exon, and as much as 84% of pepper [16]. Previous work showed that the origin of the plant GRAS gene was obtained from the prokaryotic genome through horizontal gene transfer, and subsequent replication events occurred in evolutionary history [43].

Our pairwise comparison and site-specific analysis results showed that the evolution of GRAS genes in plants after replication was limited by the choice of purification. In studies of molecular evolution, the ratio of nonsynonymous substitution per site (Ka) to synonymous substitution rate per site (Ks) which shows N- and C-terminal functional domains is widely used as an indicator of selection pressure acting on protein-coding genes (Ka/Ks). While Ka/Ks <1 indicates functional limitations due to purification or negative selection [44]. These might explain why there are a large number of intron-free GRAS genes in the switchgrass genome.

### 4.2. Characteristics of PvGRAS genes’ structure and distribution

Gene structure analysis shown that seven PvGRAS had more than two introns, most of genes contained zero, one or two introns, in detail, a total of 67 (50%) GRAS genes had no introns. This exon-intron structural characteristic of PvGRAS genes was similar to this family in other species [15,16,19]. Although all PvGRAS proteins contained GRAS conserved motifs, PvGRAS members showed many differences in chemical-physical characteristics. And the DELLA structure was only found in the DELLA subgroup. The amino acid differences in the non-conserved regions of PvGRAS members might play a key role in the differences in the physicochemical properties of the PvGRAS gene family. This meant that PvGRAS proteins with different physical and chemical properties might play different roles in resisting external stress.

144 PvGRAS genes were distributed to all eight paralogous pairs chromosomes of switchgrass with a clear nonrandom distribution, they were distributed from chromosome 1 to chromosome 9 except for chromosome 6. The location of PvGRAS genes was basically the same referred to PvNAC on each pair of chromosomes, both from chromosome 1 to chromosome 9 [45], while there were no GRAS genes in chromosome 6. It was supposed that some PvGRAS maybe lost or horizontally transferred during millions of years of evolution. The functions of most GRAS subfamily transcription factors become clearer in many plants [46].

### 4.3. The role of GRAS in abiotic stress

Most of the PvGRAS proteins have transcriptional regulatory activity and protein binding function [47,48]. Protein-protein interaction (PPI) was constructed to explore the potential interaction relationship. GAI (PvGRAS2, 6, 62, 73, 115, 116, 134 and 135) negatively regulates the GA response and participates in gibberellin-mediated signaling. It is a member of the DELLA protein and can inhibit cell proliferation and expansion, thereby driving plant growth. GAI may be involved in reducing ROS accumulation in stress responses by up-regulating the transcription of superoxide dismutase. SHR (PvGRAS13, 21, 123 and 140) is referred to radial pattern formation in roots and is required for normal shoot gravitropism, which could directly control the transcription of SCR (PvGRAS23, 33, 44, 49, 50, 51 and 85) and of MGP, RLK, TRI, NUC, and SCL3 when associated with SCR. GID1A, GID1B, and GID1C interact with specific DELLA proteins that are required for GA signaling that controls root growth, seed germination, stem elongation and flower development. Compared with other DELLA proteins, RGA1 (PvGRAS31 and 68) is the most sensitive to GA application, whereas GAI (PvGRAS2, 6, 62, 73, 115, 116, 134 and 135) is less sensitive to GA. RGL1 (PvGRAS89 and 106), RGL2 (PvGRAS39) and RGL3 (PvGRAS70) probably acts by participating in large multiprotein complexes that repress transcription of GA- inducible genes.

More than 30 GRAS proteins have protein binding function, suggesting that these genes are major transcription factors during its growth and development [49]. In addition, previous research had demonstrated that GRAS genes, such as LAS and SCL subfamily, played vital role in responses to abiotic stresses [50–53]. Several hormone and environment response elements, such as GA, auxin, light and temperature, which anticipated the growth and development of plants, were found in the promoter regions [43,54]. DELLA proteins are regarded as the key regulators of gibberellin (GA) and light signal transduction pathways [55]. These cis-element analysis results could supply clues for GRAS function research and provide evidence for the resilience of GRAS.

In order to understand the GRAS gene family in the development and adaption of switchgrass, we conducted multi-dimensional exploration, including miRNA prediction, tissue specific expression, GO enrichment analysis and qPCR verification. A variety of miRNAs were predicted that can target the PvGRAS gene family. Part of miRNA (miR164, 171, etc) have been extensively studied. Previous studies have shown that the development of tissues such as florets, branches, lateral branch growth and adventitious roots are closely related to miR164 negative regulatory the expression level of genes [56–59]. miR164 might regulate the growth of inflorescence by inhabiting the expression of PvGRAS66. PvGRAS7 and PvGRAS8 are the target genes of miR171. There may be redundant functions in the regulatory network of meristem maintenance involved in miRNA171 [60–63]. The expression level of PvGRAS3, PvGRAS140 and PvGRAS58 were low in all tissues, The role of these three GRAS genes might be to compete with PvGRAS66. PvGRAS7 and PvGRAS8 for miR171, thereby regulating the stress resistance behavior of plants. During the tissue specific analysis, the greatly various was observed in expression levels among different GRAS genes, such variation was observed in other plants [15]. An extensive analysis was performed in different tissues and abiotic stress to explore the role of GRAS. Our data shown that fourteen GRAS genes (PvGRAS7, 8, 49, 121 (leaf), 10, 12, 17, 20, 65, 29, 66, 81, 89, 105) had higher expression values and might play a more critical role in the development of tissue. The GO enrichment analysis shown that PvGRAS49 was related to root, leaf development and bundle sheath cell fate specification, the expression of PvGRAS121 was link to the growth of leaf, additionally. Several hormone response items (ethylene, abscisic acid, gibberellin and gibberellic acid), some tissues and organs’ development items (secondary shoot formation, seed dormancy process and seed germination), some items closely related to plant survival (gravitropism and hyperosmotic salinity response) have also been enriched. The qPCR results proved that PVGRAS genes shown cross-talking in the adaptation against salt and drought stress. PVGRAS17 and PVGRAS103 might be used for drought and salt tolerance breeding respectively. In summary, all these results shown that PvGRAS gene family not only participated the growth of individual, but in the adaption to environment stress.

## 5. Conclusion

In conclusion, a comprehensively analysis of the switchgrass GRAS family was made on identification and characterization in this study. A total of 144 GRAS genes in switchgrass were identified and could be categorized into 8 subfamilies. The family has a complex evolutionary history in the switchgrass genome as evidenced by gene structure and motif composition. In a word, this study was the first report to analyze PvGRAS genes roundly in switchgrass. All these data provided fundamental references for elucidating the molecular mechanisms of GRAS-mediated plant growth and development in switchgrass. The current results would be helpful to use of switchgrass GRAS at a deeper level of genetic inheritance research.

## Supplementary Material

Supplemental Material

## Highlight

144 GRAS genes were identified from switchgrass and could be divided into eight subfamilies. GRAS genes played vital role in the adaption to salt and drought stress. GRAS gene family has a complex evolutionary history in switchgrass genome.

## References

[1] Keshwani DR, Cheng JJ. Switchgrass for bioethanol and other value-added applications: a review. Bioresour Technol. 2009;100(4):1515–1523. 18976902 10.1016/j.biortech.2008.09.035

[2] Lee M, Kim B, Song S, et al. Large-scale analysis of the gras gene family in arabidopsis thaliana. Plant Mol Biol. 2008;67(6):659–670. 18500650 10.1007/s11103-008-9345-1

[3] Gunter LE, Tuskan GA, Wullschleger SD. Diversity among populations of switchgrass based on rapd markers. Crop Sci. 1996;36(4):1017–1022.

[4] Guo P, Wen J, Yang J, et al. Genome-wide survey and expression analyses of the gras gene family in brassica napus reveals their roles in root development and stress response. Planta. 2019;250(4):1051–1072. 31161396 10.1007/s00425-019-03199-y

[5] Karam B, Singh A, Rhonda C, et al. Transcription factors in plant defense and stress responses. Curr Opin Plant Biol. 2002;5(5):430–436. 12183182 10.1016/s1369-5266(02)00289-3

[6] Frazier TP, A. PN, Fuliang X, et al. Identification, characterization, and gene expression analysis of nucleotide binding site (nb)-type resistance gene homologues in switchgrass. BMC Genomics. 2016;17(1):892. 27821048 10.1186/s12864-016-3201-5 PMC5100175

[7] Rinerson ED, Na S, Donze-Reiner P, et al. The WRKY transcription factor family and senescence in switchgrass. BMC Genomics. 2015;16(1):912. 26552372 10.1186/s12864-015-2057-4 PMC4640240

[8] Pysh LD, Wysocka-Diller JW, Camilleri C, et al. The GRAS gene family in arabidopsis: sequence characterization and basic expression analysis of the scarecrow-like genes. Plant J Cell Mol Biol. 1999;18(1):111–119. 10.1046/j.1365-313x.1999.00431.x 10341448

[9] Peng J, Carol P, Richards DE, et al. The arabidopsis gai gene defines a signaling pathway that negatively regulates gibberellin responses? Genes Dev. 1997;11(23):3194–3205. 9389651 10.1101/gad.11.23.3194 PMC316750

[10] Sun X, Jones WT, Rikkerink AEHA. Gras proteins: the versatile roles of intrinsically disordered proteins in plant signalling. Biochem J. 2012;442(1):1–12. 22280012 10.1042/BJ20111766

[11] Pan L, Bin Z, Tongbing S, et al. BrLAS, a GRAS transcription factor from brassica rapa, is involved in drought stress tolerance in transgenic arabidopsis. Front Plant Sci. 2018;9. DOI:10.3389/fpls.2018.01792. PMC6291521 30574156

[12] Guo Z, Gao Y, Cao X, et al. Phytoremediation of Cd and Pb interactive polluted soils by switchgrass (panicum virgatum l.). Int J Phytoremediation. 2019;1–11. DOI:10.1080/15226514.2019.1644285. 31342773

[13] Cai H, Chen Y, Zhang M, et al. A novel GRAS transcription factor, ZmGRAS20, regulates starch biosynthesis in rice endosperm. Physiol Mol Biol Plants. 2017;23(1):143–154. 28250591 10.1007/s12298-016-0404-9 PMC5313408

[14] Liu Y, Wei H, Zhiqiang X, et al. Overexpression of SIGRAS40 in tomato enhances tolerance to abiotic stresses and influences auxin and gibberellin signaling. Front Plant Sci. 2017;8:1659. 29018467 10.3389/fpls.2017.01659 PMC5622987

[15] Huang W, Xian Z, Kang X, et al. Genome-wide identification, phylogeny and expression analysis of gras gene family in tomato. BMC Plant Biol. 2015;15(1):209–226. 26302743 10.1186/s12870-015-0590-6 PMC4549011

[16] Liu B, Sun Y, Xue J, et al. Genome-wide characterization and expression analysis of gras gene family in pepper (capsicum annuum). *Peerj* l. 2018;6(e4796). DOI:10.7717/peerj.4796 PMC5983004 29868257

[17] Liu X, Widmer A. Genome-wide comparative analysis of the gras gene family *Inpopulus, Arabidopsis* and rice. Plant Mol Biol Rep. 2014;32(6):1129–1145.

[18] Lu J, Wang T, Xu Z, et al. Genome-wide analysis of the gras gene family in Prunus mume. Mol Genet Genomic. 2014;290(1):303–317. 10.1007/s00438-014-0918-1 25245166

[19] Quan S, Niu J, Zhou L, et al. Open genome-wide identification, classification, expression and duplication analysis of gras family genes in juglans regia l. Sci Rep. 2019;9. DOI:10.1038/s41598-019-48287-x. 31406208 PMC6691012

[20] Sun X, Xue B, Jones WT, et al. A functionally required unfoldome from the plant kingdom: intrinsically disordered N-terminal domains of gras proteins are involved in molecular recognition during plant development. Plant Mol Biol. 2011;77(3):205–223. 21732203 10.1007/s11103-011-9803-z

[21] Chen C, Xia R, Chen H, et al. TBtools, a toolkit for biologists integrating various hts-data handling tools with a user-friendly interface. Biorxiv. 2018;289660. DOI:10.1101/289660

[22] Liu M, Fu Q, Ma Z, et al. Genome-wide investigation of the mads gene family and dehulling genes in tartary buckwheat (fagopyrum tataricum). Planta. 2019;5(5):1301–1318. 10.1007/s00425-019-03089-3 30617544

[23] Wang S, Bai Y, Li P, et al. Physiological and molecular plant pathology. Physiol Mol Plant Pathol. 2019;108:1–12.

[24] Wang S, Zhang N, Zhu X, et al. Identification and expression analysis of stgras gene family in potato (solanum tuberosum) l. Computational Biology and Chemistry. 2019;80:195–205. 10.1016/j.compbiolchem.2019.03.020 30978571

[25] Chen Y, Zhu P, Wu S, et al. Identification and expression analysis of gras transcription factors in the wild relative of sweet potato ipomoea trifida. BMC Genomics. 2019;20(1):1–12. 31783728 10.1186/s12864-019-6316-7 PMC6884806

[26] Li X, Guo C, Ahmad S, et al. Systematic analysis of myb family genes in potato and their multiple roles in development and stress responses. Biomolecules. 2019;9(8):317–337. 10.3390/biom9080317 PMC6723670 31366107

[27] Cao Y, Yahui H, Dahui L, et al. MYB transcription factors in chinese pear (Pyrus bretschneideri Rehd.): genome-wide identification, classification, and expression profiling during fruit development. Front Plant Sci. 2016;7:577. 27200050 10.3389/fpls.2016.00577 PMC4850919

[28] Li X, Ahmad S, Guo, C, et al. Identification and characterization of lrr-rlk family genes in potato reveal their involvement in peptide signaling of cell fate decisions and biotic/abiotic stress responses. Cells. 2018;7(9):120. 10.3390/cells7090120 PMC6162732 30150583

[29] Szklarczyk D, Morris JH, Cook H, et al. The string database in 2017: quality-controlled protein–protein association networks, made broadly accessible. Nucleic Acids Res. 2017;45(D1):D362–D368. 27924014 10.1093/nar/gkw937 PMC5210637

[30] Otasek D, Morris JH, Bouças J, et al. Cytoscape automation: empowering workflow-based network analysis. Genome Biol. 2019;20(1):185. 31477170 10.1186/s13059-019-1758-4 PMC6717989

[31] Librado P, Rozas J. DnaSP v5: a software for comprehensive analysis of DNA polymorphism data. Bioinformatics. 2009;25:1451–1452. 19346325 10.1093/bioinformatics/btp187

[32] Lescot M, Lescot, and M. PlantCARE, a database of plant cis-acting regulatory elements and a portal to tools for in silico analysis of promoter sequences. Nucleic Acids Res. 2002;30(1):325–327. 11752327 10.1093/nar/30.1.325 PMC99092

[33] Zhang JY, Lee YC, Torres-Jerez I, et al. Development of an integrated transcript sequence database and a gene expression atlas for gene discovery and analysis in switchgrass (Panicum virgatum L.). Plant J. 2013;74(1):160–173. 10.1111/tpj.12104 23289674

[34] Zhang S, Xu R, Luo X, et al. Genome-wide identification and expression analysis of MAPK and MAPKK gene family in malus domestica. Gene. 2013;531(2):377–387. 23939467 10.1016/j.gene.2013.07.107

[35] Li YF, Wang Y, Tang Y, et al. Transcriptome analysis of heat stress response in switchgrass (panicum virgatum L.). BMC Plant Biol. 2013;13(1):153. 24093800 10.1186/1471-2229-13-153 PMC3851271

[36] Tian C, Wan P, Sun S, et al. Genome-wide analysis of the gras gene family in rice and arabidopsis. Plant Mol Biol. 2004;54(4):519–532. 15316287 10.1023/B:PLAN.0000038256.89809.57

[37] Guo Y, Hongyu W, Xiang L, et al. Identification and expression of gras family genes in maize (zea mays l.). PloS One. 2017;12(9):e185418. 10.1371/journal.pone.0185418 PMC5619761 28957440

[38] Grimplet J, Agudelo-Romero P, Teixeira RT, Martinez-Zapater JM, Fortes AM. Structural and functional analysis of the gras gene family in grapevine indicates a role of gras proteins in the control of development and stress responses. Front Plant Sci. 2016;7:353. 27065316 10.3389/fpls.2016.00353 PMC4811876

[39] Lu J, Wang T, Xu Z, et al. Genome ‑ wide analysis of the gras gene family in prunus mume. Mol Genet Genomics. 2015;290(1):303–307. 25245166 10.1007/s00438-014-0918-1

[40] Huang S, Su X, Haselkorn R, et al. Evolution of switchgrass (panicum virgatum l.) Based on sequences of the nuclear gene encoding plastid acetyl-COA carboxylase. Plant Sci. 2003;164(1):49.

[41] Yuan S, Xu B, Zhang J, et al. Comprehensive analysis of CCCH-type zinc finger family genes facilitates functional gene discovery and reflects recent allopolyploidization event in tetraploid switchgrass. BMC Genomics. 2015;16(1):129. 25765300 10.1186/s12864-015-1328-4 PMC4352264

[42] Okada M, Lanzatella C, Saha MC, et al. Complete switchgrass genetic maps reveal subgenome collinearity, preferential pairing and multilocus interactions. Genetics. 2010;185(3):745–760. 20407132 10.1534/genetics.110.113910 PMC2907199

[43] Kepinski S. Integrating hormone signaling and patterning mechanisms in plant development. Curr Opin Plant Biol. 2006;9(1):28–34. 16325457 10.1016/j.pbi.2005.11.001

[44] Chi Y, Cheng Y, Jeevanandam V, et al. Expansion mechanisms and functional divergence of the glutathione s-transferase family in sorghum and other higher plants. DNA Res. 2010;1. DOI:10.1093/dnares/dsq031. PMC3041506 21169340

[45] HaidongYan Z, YuntianYe A, Chen J, et al. Genome-wide survey of switchgrass nacs family provides new insights into motif and structure arrangements and reveals stress-related and tissue-specific NACs. Sci Rep. 2017;7(1):3056. 28596552 10.1038/s41598-017-03435-z PMC5465074

[46] Zentella R, Zhang Z, Park M, et al. Global analysis of DELLA direct targets in early gibberellin signaling in Arabidopsis. Plant Cell. 2007;19(10):3037–3057. 17933900 10.1105/tpc.107.054999 PMC2174696

[47] Xu W, Zexi C, Naeem A, et al. Genome-wide identification, evolutionary analysis, and stress responses of the GRAS gene family in castor beans. Int J Mol Sci. 2016;17(7):1004. 10.3390/ijms17071004 PMC4964380 27347937

[48] Zhang BJ, Liu ZY, Chen E, et al. Genome-wide analysis of GRAS transcription factor gene family in Gossypium hirsutum L. BMC Genomics. 2018;19(1). DOI:10.1186/s12864-018-4722-x PMC5944045 29743013

[49] Jérô M, Patricia G, T.r. AT, et al. Structural and functional analysis of the gras gene family in grapevine indicates a role of gras proteins in the control of development and stress responses. Front Plant Sci. 2016;7:353. 27065316 10.3389/fpls.2016.00353 PMC4811876

[50] Day B, Shibuya N, Minami E. Identification and characterization of two new members of the GRAS gene family in rice responsive to N-acetylchitooligosaccharide elicitor. Biochim Biophys Acta. 2003;1625(3):261–268. 12591613 10.1016/s0167-4781(02)00626-7

[51] Hong-Shuang M, Liang D, Shuai P, et al. The salt- and drought-inducible poplar GRAS protein SCL7 confers salt and drought tolerance in Arabidopsis thaliana. J Exp Bot. 2010;61(14):4011–4019. 20616154 10.1093/jxb/erq217 PMC2935874

[52] Xu K, Chen S, Tianfei L, et al. OsGRAS23, a rice GRAS transcription factor gene, is involved in drought stress response through regulating expression of stress-responsive genes. BMC Plant Biol. 2015;15. DOI:10.1186/s12870-015-0532-3 26067440 PMC4465154

[53] Yang M, Yang Q, Tingdong F, et al. Overexpression of the Brassica napus BnLAS gene in Arabidopsis affects plant development and increases drought tolerance. Plant Cell Rep. 2010;30(3):373–388. 20976458 10.1007/s00299-010-0940-7

[54] Kami C, Lorrain SV, Hornitschek P, et al. Light-regulated plant growth and development. Curr Top Dev Biol. 2010;91:29–66. 20705178 10.1016/S0070-2153(10)91002-8

[55] Fukazawa J, Teramura H, Murakoshi S, et al. DELLAs function as coactivators of gai-associated factor1 in regulation of gibberellin homeostasis and signaling in arabidopsis. Plant Cell. 2014;26(7):2920–2938. 25035403 10.1105/tpc.114.125690 PMC4145123

[56] Chen S. Overexpression of BpCUC2 influences leaf shape and internode development in betula pendula. Int J Mol Sci. 2020;20:19. 10.3390/ijms20194722 PMC6801603 31548512

[57] Geng Y, Jian C, Xu W, et al. miR164-targeted TaPSK5 encodes a phytosulfokine precursor that regulates root growth and yield traits in common wheat (Triticum aestivum L.). Plant Mol Biol. 2020;104(6):615–628. 10.1007/s11103-020-01064-1 32968950

[58] Li J, Zhang H, Zhu J, et al. Role of miR164 in the growth of wheat new adventitious roots exposed to phenanthrene. Environ Pollut. 2021;284:117204. 33910135 10.1016/j.envpol.2021.117204

[59] Zhan J, Chu Y, Wang Y, et al. The miR164-GhCUC2-GhBRC1 module regulates plant architecture through abscisic acid in cotton. Plant Biotechnol J. 2021. DOI:10.1111/pbi.13599 PMC8428825 33960609

[60] Beheshti H, Christoph Strotbek M, Arif A, et al. PpGRAS12 acts as a positive regulator of meristem formation in Physcomitrium patens. Plant Mol Biol. 2021;1–13. DOI:10.1007/s11103-021-01125-z PMC8648639 33598827

[61] Curaba J, Talbot M, Zhongyi L, et al. Over-expression of microRNA171 affects phase transitions and floral meristem determinancy in barley. BMC Plant Biol. 2013;13(1):6. 23294862 10.1186/1471-2229-13-6 PMC3547705

[62] Huang W, Peng S, Xian Z, et al. Overexpression of a tomato miR171 target gene SlGRAS24 impacts multiple agronomical traits via regulating gibberellin and auxin homeostasis. Plant Biotechnol J. 2016;15. DOI:10.1111/pbi.12646. PMC5362688 27712008

[63] Tong A, Yuan Q, Shu W, et al. Altered accumulation of osa-miR171b contributes to rice stripe virus infection by regulating disease symptoms. J Exp Bot. 2017;68(15):4357–4367. 28922766 10.1093/jxb/erx230 PMC5853540

